# Proteomics-Based Identification of Diagnostic Biomarkers Related to Risk Factors and Pathogenesis of Ischemic Stroke

**DOI:** 10.3390/diagnostics10050340

**Published:** 2020-05-25

**Authors:** Jiyeong Lee, Arum Park, Sora Mun, Hyo-Jin Kim, Hyunsong Son, Hyebin Choi, Doojin Kim, Soo Joo Lee, Jae Guk Kim, Hee-Gyoo Kang

**Affiliations:** 1 Department of Biomedical Laboratory Science, School of Medicine, Eulji University, Daejeon 34824, Korea; bjssw@naver.com; 2 Department of Senior Healthcare, BK21 Plus Program, Graduate School, Eulji University, Seongnam 13135, Korea; sksskdi5959@naver.com (A.P.); sora6456@naver.com (S.M.); hyojin0222@naver.com (H.-J.K.); latias94@naver.com (H.S.); chb1996@naver.com (H.C.); 3 Department of Laboratory Medicine, Seongnam Central Hospital, Seongnam 13161, Korea; djkingdom@hanmail.net; 4 Department of Neurology, Eulji University Hospital, School of Medicine, Eulji University, Daejeon 35233, Korea; sjoolee@eulji.ac.kr (S.J.L.); jgkim@eulji.ac.kr (J.G.K.); 5 Department of Biomedical Laboratory Science, School of Medicine, Eulji University, Seongnam 13135, Korea; 6 Seongnam Senior Industry Innovation Center, Eulji University, Seongnam 13503, Korea

**Keywords:** ischemic stroke, plasma biomarker, proteomics, diagnosis, individual analysis

## Abstract

Ischemic stroke is caused by blood clot formation and consequent vessel blockage. Proteomic approaches provide a cost-effective alternative to current diagnostic methods, including computerized tomography (CT) scans and magnetic resonance imaging (MRI). To identify diagnostic biomarkers associated with ischemic stroke risk factors, we performed individual proteomic analysis of serum taken from 20 healthy controls and 20 ischemic stroke patients. We then performed SWATH analysis, a data-independent method, to assess quantitative changes in protein expression between the two experimental conditions. Our analysis identified several candidate protein biomarkers, 11 of which were validated by multiple reaction monitoring (MRM) analysis as novel diagnostic biomarkers associated with ischemic stroke risk factors. Our study identifies new biomarkers associated with the risk factors and pathogenesis of ischemic stroke which, to the best of our knowledge, were previously unknown. These markers may be effective in not only the diagnosis but also the prevention and management of ischemic stroke.

## 1. Introduction

Ischemic stroke, which may cause permanent disability [1], is caused by various conditions, such as high blood pressure [2], diabetes [3], and heart problems [4], and can manifest suddenly following blocked or burst blood vessels [5]. Hence, ischemic stroke prevention is most effective when controlling its direct or indirect associated risk factors. For example, individuals with hypertension are twice as likely to develop ischemic stroke than their healthy counterparts, and diabetic individuals are more than twice as likely to develop ischemic stroke as compared to healthy individuals. However, heart disease remains a major cause of cerebral infarction.

Biomarkers include factors with the capacity to objectively predict therapeutic responses or discriminate between the physiological and pathological conditions [6] and are categorized based on their function or purpose. The developmental processes for novel biomarkers fall into two main categories: discovery and validation processes [7]. The discovery process identifies candidate biomarkers, wherein most experiments are processed using a label-free quantification method, which is one of the most commonly used methods for comparing different groups after pooling samples [8].

However, pooled samples may cause false-positive or false-negative results, as they do not reflect the condition of a specific individual. Therefore, individual analysis of each sample, to represent individual status for identifying more efficient and accurate diagnostic biomarkers, is required. Previous studies that have performed individual analyses have used small sample sizes [9]; however, to find a sensitive and specific biomarker, individual analysis in large sample cohorts is warranted.

In this study, for more effective diagnosis and treatment options, we have identified candidate diagnostic markers associated with known risk factors for ischemic stroke. We analyzed serum samples from healthy controls and ischemic stroke patients, divided the samples into a discovery set and a validation set, and processed individual serum samples (i.e., without pooling). For the discovery process, SWATH-MS was used, while multiple reaction monitoring (MRM) analysis was used for validation.

## 2. Results

### 2.1. Identification and Quantification of Serum Proteins

To identify biomarkers for early diagnosis of ischemic stroke, serum samples from 20 healthy controls and 20 ischemic stroke patients were individually analyzed. We identified 587 ischemic stroke-related proteins (global FDR <1%) using Proteinpilot v5.0 (Supplementary Table S1). Next, through SWATH acquisition using the DIA method, we identified and quantified 399 differentially expressed proteins and confirmed that the following proteins were not represented in any of the healthy control individuals: tropomyosin alpha-4 chain 9 (P67936), latent-transforming growth factor beta-binding protein 1 (Q14766), insulin-like growth factor-binding protein 7 (Q16270), and osteopontin (P10451). We then performed principal component analysis (PCA) for the classification of healthy controls and ischemic stroke patients. Supplementary Figure S1a shows proteins used to classify healthy controls and ischemic stroke patients; the middle dense, deep purple scatters represent proteins that were not significantly different in the two groups, while the light purple scatters represent proteins differentially expressed in both the groups. The healthy control group and ischemic stroke patient group were shown to be clustered and well differentiated (Supplementary Figure S1b).

### 2.2. Differentially Expressed Proteins in Serum Proteins from Healthy Control and Ischemic Stroke Patients

**Figure 1:**
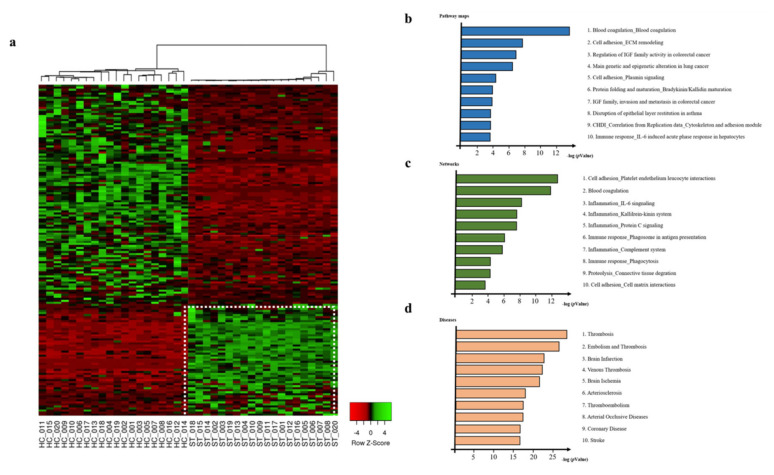
Analysis of 163 differentially expressed proteins compared with healthy controls and ischemic stroke patients. (**a**) Hierarchical clustered heat map shows 163 significantly differentially expressed proteins pattern. Clustering analysis was performed by Pearson distance method for normalized intensity. Enrichment analysis by GeneGo represents (**b**) top 10 significant pathway maps, (**c**) networks, and (**d**) diseases. The ranking is based on the value of p-value. HC (healthy control), ST (ischemic stroke patient).

We found that 163 out of the 399 proteins identified by SWATH analysis showed more than twofold differential expression in patients with ischemic stroke (Figure 1a). To confirm the statistical significance of the differentially expressed proteins, we applied Benjamini–Hochberg correction to obtain an FDR-adjusted corrected *p*-value; the cutoff value of the corrected *p*-value was 0.05, suggesting that 5% of the differentially expressed proteins could be false positive. Functional analysis of the differentially expressed proteins using GeneGo software identified the top 10 pathway maps associated with ischemic stroke, including blood coagulation, cell adhesion, and immune response (Figure 1b). The network analysis also showed an association of the differentially expressed proteins with cell adhesion, blood coagulation, and immune response (Figure 1c). We also identified that thrombosis, embolism, and brain infarction were highly ranked among the diseases associated with the differentially expressed proteins (Figure 1d). Thus, we speculated that the differentially regulated proteins could be closely associated with ischemic stroke pathogenesis.

### 2.3. Selection of Potential Biomarker Candidates

**Figure 2:**
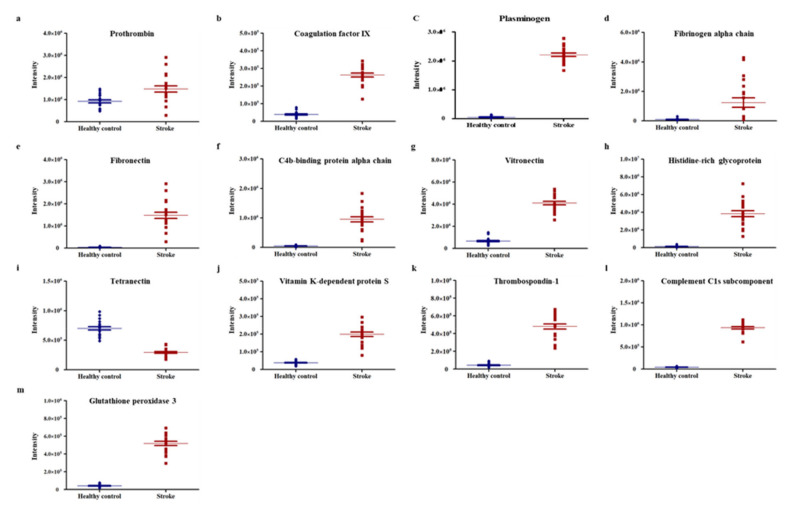
Identification of 13 proteins with high tendency in healthy controls and ischemic stroke patients. (**a**–**m**) The 13 high-trend proteins in each sample were identified by dot plot using MarkerView software. Line in the middle of the dots represents mean value.

Among the 163 differentially expressed proteins, 13 were selected as potential biomarker candidates (Figure 2). The distribution of these proteins, confirmed by dot plot using the MarkerView software, was found to represent distinct differences between the two groups. Further, the intensity between individuals of a group also showed a similar pattern. The use of STRING (Search Tool for the Retrieval of Interacting Genes/Proteins) software (http://string-db.org/) for identification of protein–protein interactions between the 13 candidate proteins revealed that fibronectin (FN1), vitamin K-dependent protein S (PROS1), plasminogen (PLG), thrombospondin 1 (THBS-1), histidine-rich glycoprotein (HRG), fibrinogen alpha chain (FGA), and prothrombin (F2) were primarily associated with other proteins and located in the central area of the network. Additionally, C4b-binding protein alpha chain (C4BP4), complement C1s subcomponent (C1S), vitronectin (VTN), coagulation factor IX (F9), and tetranectin (CLEC3B) were associated with other proteins and located in the outer area. All the proteins had associations with other proteins, except for glutathione peroxidase 3 (GPx-3) (Supplementary Figure S2).

### 2.4. MRM Verification of Candidate Marker Proteins

**Figure 3:**
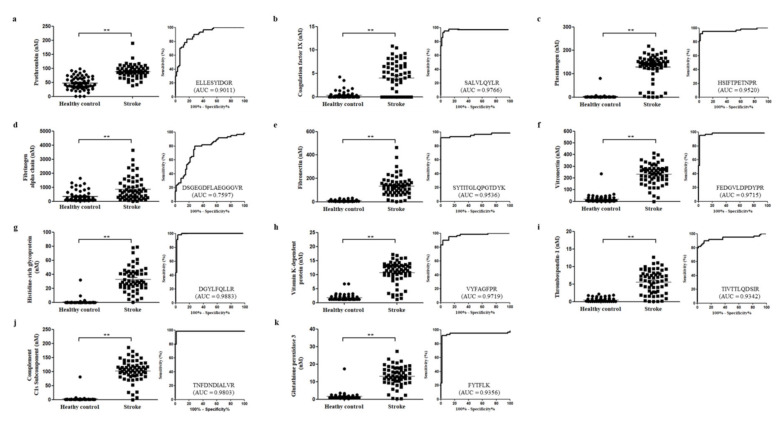
Scatter plots and ROC curves. The figures show dot plots and ROC curves of 11 candidate proteins in comparison with ischemic stroke and healthy control as MRM verification results. (**a**) prothrombin, (**b**) coagulation factor, (**c**) plasminogen, (**d**) fibrinogen alpha chain, (**e**) fibronectin, (**f**) vitronectin, (**g**) histidine-rich glycoprotein, (**h**) vitamin K-dependent protein, (**i**) thrombospondin 1, (**j**) complement C1s subcomponent, (**k**) glutathione peroxidase 3. An ROC curve is a plot of sensitivity on the y-axis against (100−specificity)% on the x-axis at all possible cut-points. AUC value was shown inside the ROC curves with sensitivity and specificity. ** *p* <0.01.

**Figure 4:**
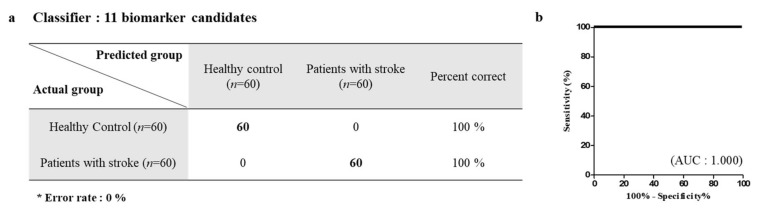
Selected biomarker candidates in healthy controls and ischemic stroke patients were analyzed by logistic analysis. (**a**) Classification tables using 11 biomarker candidates. Classification accuracy was 100% in two groups. (**b**) AUC curves of 11 markers were plotted. The 60 healthy controls and the 60 ischemic stroke patients were used for logistic analysis.

The selected 13 proteins were quantified using MRM; one peptide per protein was used for quantification. The peptide with the highest intensity and FDR <1 was selected to represent the abundance of the candidate protein. Among the 13 proteins, 11 proteins exhibited strong discriminatory efficacy by ROC analysis, wherein the area under the curve (AUC) of 10 proteins was >0.90, while that of the fibrinogen alpha chain was >0.70. In addition, the two proteins that had AUC <0.70 were excluded as the final potential candidates. AUC <0.70 indicated weak classification model. The AUC values for F2, F9, PLG, FGA, VTN, HRG, PROS1, THBS-1, C1S, and GPx-3 were 0.911, 0.9766, 0.9520, 0.7597, 0.9715, 0.9883, 0.9719, 0.9342, 0.9803, and 0.9356, respectively (Figure 3). Logistic analysis for the evaluation of the selected 11 proteins was also performed. As shown in Figure 4, the classification accuracy of the selected proteins was 100%, suggesting that the selected proteins can greatly help to distinguish ischemic stroke patients from healthy controls.

### 2.5. Relationship of Candidate Marker and Ischemic Stroke Risk Factors/Ischemic Stroke Pathogenesis

Next, we identified the relationship between risk factors and processes involved in the pathogenesis of ischemic stroke and the 11 candidate proteins. The proteins were found to be associated with at least one risk factor or one process involved in pathogenesis. F2 and F9 were associated with cardiovascular disease (CVD) and diabetes, whereas PLG was only related to apoptosis, which is involved in ischemic stroke pathogenesis. FGA was associated with three risk factors (hypertension, CVD, and diabetes) and two processes involved in pathogenesis (immune response and apoptosis). FN1 was associated with two risk factors (CVD and diabetes) and one pathogenesis process (immune response). VTN was associated with CVD and diabetes as well as immune response and apoptosis. HRG and PROS1 were associated with CVD and high cholesterol, respectively, and both were associated with immune response and apoptosis. THBS-1 was associated with all the risk factors and pathogenesis processes, whereas C1S was found to be related to immune response. Finally, GPx-3 was associated with hypertension, CVD, and diabetes as well as two pathogenesis processes, oxidative stress and apoptosis (see Table 1).

## 3. Discussion

Ischemic stroke is caused by blood clots blocking blood vessels. Blood clotting results from various causes, including arteriosclerosis and cardiac embolism, and is associated with blood vessel damage caused by a myriad of risk factors, which promote the activity of procoagulants involved in blood coagulation [10]. F9, F2, and FGA, which were selected as potential biomarker candidates in this study, are the major factors involved in coagulation. F2 and FGA are precursors of thrombin and fibrin [11]; F2 is converted to thrombin by activated coagulation factor X, which is activated by F9 [12]. Thrombin converts fibrinogen to fibrin, which forms blood clots by aggregating to platelets [13]. In previous studies, prothrombin gene mutation and an increase in F9 and FGA production have been reported to be closely associated with stroke risk factors, such as CVD [14] and diabetes [15]. In CVD and diabetes patients, cholesterol deposits increase in blood vessel walls, thus narrowing blood vessels and generating blood clots, which interrupt blood flow [16]. These blood clots ultimately cause an ischemic stroke by blocking blood vessels. Therefore, the factors involved in blood clotting are maintained at high levels in these diseases. According to a previous study, prothrombin gene mutation and F9 and FGA expression are increased in stroke patients [17]. This observation was confirmed in our study.

FGA is associated with stroke pathogenesis [17], including oxidative stress, immune response, and apoptosis [18]. Recent findings suggest that fibrinogen promotes neutrophil activation by specifically binding to the alpha-subunit of CD11b/CD18 on the surface of neutrophils, which plays a major role in ischemic brain injury. Various cytokines activate neutrophils; activated neutrophils migrate to the brain through interactions with endothelial cells, exacerbating the pathogenesis of stroke by releasing reactive oxygen species (ROS), proteases, and cytokines that disrupt the blood–brain barrier [19].

FN1 and VTN are glycoproteins that are involved in blood coagulation by binding to fibrin with the help of coagulation factor XIII, as well as forming platelet aggregates by binding platelets [20]. VTN inhibits tissue plasminogen activator (tPA), which plays a pivotal role in fibrinolysis, which dissolves blood clots. It is also involved in the formation of the stable plasminogen activator inhibitor-1 (PAI-1) conformation by binding with a PAI-1, thereby promoting the formation of blood clots. The interaction of PAI-1 with VTN has been reported to promote thrombosis [21]. FN1 and VTN, like other factors involved in the coagulation process, are known to increase not only in stroke patients [22] but also in CVD [23] and diabetes [24] patients. Our results showed that FN1 and VTN levels were higher in ischemic stroke patients than in healthy controls, which was consistent with previous studies. We also found that FGA and VTN were associated with immune response. FN1 and VTN activate microglia when brain damage occurs due to conditions such as ischemic stroke. When microglia, immune response modulators in damaged brain tissue, are activated, they secrete proinflammatory cytokines, such as interleukin 6 (IL-6) and tumor necrosis factor (TNF)-α. Studies have indicated that microglia damage neurons in a TLR-4-dependent manner in early ischemic stroke, while inhibition of microglia activation reduces brain infarction and blood–brain barrier (BBB) leakage. It has also been reported that microglia activated by FN1 and VTN promote the progression of ischemic stroke [25].

HRG is synthesized by immune cells, such as monocytes, macrophages, and megakaryocytes, and is present in plasma and platelets [26]. HRG is associated with the immune response and coagulation process through a high-affinity interaction with fibrinogen or fibrin [27]. In a previous proteomic study, HRG was reported to be higher in the platelets of stroke patients than in those of healthy controls. Moreover, platelets are reportedly activated during stroke while HRG binds to the activated platelets surface [22]. Our study also demonstrated that HRG was highly expressed in ischemic stroke patients.

THBS-1, a major granule protein of platelets, binds to fibrinogen, FN1, laminin, and collagen and influences various processes, such as inflammatory response, apoptosis, and angiogenesis [28]. THBS-1 has also been shown to be upregulated in various diseases caused by brain damage [29], during which it is released from damaged brain tissue into the blood from cerebrospinal fluid [30]. THBS-1 is released from platelets to repair damaged tissue and promote platelet aggregation through interaction with platelets, possibly forming interplatelet cross-bridges through interaction with fibrinogen [31]. It has also been reported that THBS-1 affects CAD [32] and diabetes [33]. In a previous study, THBS-1 showed higher expression in platelets of ischemic stroke patients than in platelets of healthy controls, suggesting that THBS-1 may serve as a suitable parameter for monitoring platelet activation [22]. Our data also showed that the expression of THBS-1 was higher in ischemic stroke patients than in healthy controls. Taken together, these results suggest that THBS-1 could serve as a blood biomarker for diagnosing ischemic stroke.

PLG and PROS1 have an anticoagulant activity that prevents coagulation and stimulates fibrinolysis [34]. PLG is a precursor of fibrin and plays a role in degradation of fibrin clots. Although direct association between PLG and ischemic stroke has not been shown, plasminogen activator, which converts PLG into plasmin, has been used to determine the prognosis and treatment of ischemic stroke [35], CVD [36], and diabetes [37].

PROS1 inhibits the thrombin generation by binding to coagulation factors Va and VIIIa [38]. Genetic mutation of PROS1 causes various diseases, including ischemic stroke by increasing thrombosis [39]. Our results indicate that the expression of PLG and PROS1 was elevated in ischemic stroke patients due to excessive blood clotting in ischemic stroke patients, which may be increased by the feedback mechanism that reduces blood clotting to maintain homeostasis.

Complement C1s subcomponent (C1s) is part of the classical complement pathway [40]. Previous studies have suggested that the complement system plays a vital role in the pathogenesis of ischemic stroke, while its suppression reduces brain damage [40]. C3 is primarily produced by neurons, astrocytes, and microglia during brain damage. However, increased C3 levels in ischemic stroke patients have previously been reported [41]. We observed that C1s assists in the activation of C3 and was upregulated in ischemic stroke patients. Taken together, C1s may be highly expressed in ischemic stroke patients to activate the complement system following ischemic stroke, which would act to restore brain injury by promoting C3 activation. Therefore, we predict that C1s influences ischemic stroke pathogenesis and could be used as a marker for the diagnosis of ischemic stroke.

GPx-3 is a major antioxidant enzyme and acts as an ROS scavenger during metabolism [42]. Following ischemic stroke, ROS generated by immune responses not only damage brain cells but also induce the release of inflammatory mediators through various signaling pathways [43]. Neurons undergo apoptosis when exposed to oxidative stress [44]. GPx-3 is elevated under oxidative stress [45] and is reportedly increased in ischemic stroke patients [46]. These results were confirmed in our study. Taken together, these findings indicate that GPx-3 could be a potential new diagnostic biomarker for ischemic stroke.

Herein, using specific analytical methods, we identified more accurate and specific biomarkers of ischemic stroke as compared to those identified in previous studies using pooled samples. However, certain limitations are associated with this study. One, in order to use the candidate biomarkers for diagnosis of ischemic stroke, further studies involving a larger sample size are warranted. Second, the serum samples used in this study were obtained exclusively from Korean individuals; therefore, further studies using samples from a more ethnically diverse group of individuals are essential for confirmation of the candidate proteins as global biomarkers of ischemic stroke. Third, to evaluate the effects of medication given to ischemic stroke patients on the expression pattern of serum proteins, we confirmed the type and characteristics of drugs administered to all patients enrolled in this study. All patients were treated with an antiplatelet drug, either aspirin or clopidogrel, and none were treated with a drug that could affect the coagulation pathway, such as tPA (tissue plasminogen activator). Even though neither aspirin nor clopidogrel affect the coagulation pathway associated with candidate biomarkers, they may affect the general serum proteome. These verified candidate biomarkers in the current paper need to be confirmed with samples of drug-naive patients in further studies. 

In our study, we aimed to identify potential diagnostic biomarkers with distinct differences between ischemic stroke patients and control groups, with the focus on finding markers linked to risk factors in ischemic stroke patients. As shown in Table 1, the identified biomarkers were classified in connection with each risk factor; thus, it is expected that patient health parameters can be identified by quantifying the expression pattern of markers associated with hypertension, CVD, diabetes, and high cholesterol simultaneously for ischemic stroke diagnosis. Previous studies have identified biomarkers through comparison of ischemic patients and control groups, and their marker analysis was conducted in a different manner. The results of marker discovery studies on plasma of ischemic patients presented markers, such as BNP, von Willebrand factor, ICAM-1, CRP, and interleukin-6, through biomarker research progress associated with causes of ischemic stroke, which differed from the biomarkers identified in our study [47,48,49]. Other studies reported the association of vasoactive peptide hormones, adhesion molecules, acute phase proteins, and inflammatory cytokines with ischemic stroke [50,51,52]. We think it is important to consider the association between the 11 proteins identified in this study and previously reported protein biomarkers. In addition to blood protein analysis, studies on marker discovery through RNA analysis are also being carried out [53,54,55,56]. Conducting research on gene-related markers, including RNA and DNA, along with related protein panels, would significantly improve our understanding of markers found in this study. To the best of our knowledge, this is the first study to identify high-accuracy blood biomarkers as diagnostic tools for ischemic stroke through individual analysis using label-free quantification methods. Since the candidate biomarkers not only showed distinct differential expression between ischemic stroke patients and healthy controls but were also found to be closely associated with ischemic stroke risk factors and pathogenesis, their implementation may serve to improve the diagnosis and management of ischemic stroke. Moreover, the biomarker discovery method used in this study may be applicable as a tool for identifying disease-specific biomarkers in other diseases.

## 4. Materials and Methods

### 4.1. Participants and Serum Collection

All serum samples used in this study were obtained from Eulji University Hospital Institutional Review Board located in Daejeon, South Korea (EMC 2016-03-019, 31 March 2016). We acquired samples from ischemic stroke patients and healthy subjects without documented ischemic stroke disease. Prior to blood sampling, we obtained informed consent from all participants. Samples were divided into discovery and validation sets (Supplementary Figure S3, Table S2). The sample sizes of the discovery set and the validation set were 20 and 60, respectively (Supplementary Table S2). Blood collection of all ischemic stroke patients was conducted within 10 days of the disease onset (i.e., when all patients were in the acute phase of ischemic stroke). The numbers of patients with history of ischemic stroke, hypertension, diabetes mellitus, atrial fibrillation, and smoking were 10, 32, 9, 5, and 17, respectively. The average of initial NIHSS score (score of disease severity) was 4. Blood samples were collected in vacutainers without anticoagulants and incubated at room temperature for 2 h. The blood samples were then centrifuged at 4000*× g* for 5 min to allow coagulation, after which the supernatants were collected. The serum samples were stored at −80 °C until further use.

### 4.2. Sample Preparation

Prior to analysis, low-abundance proteins (100 μg) were collected by removing highly abundant proteins that constituted approximately 99% of the total serum proteins using a multiple affinity removal system (MARS) column (human 6, 4.6 × 50 mm; Agilent Technologies, Santa Clara, CA, USA). Data-dependent acquisition (DDA)-generated spectral libraries were constructed using the pooled sample prepared from the serum samples of the 20 ischemic stroke patients and 20 healthy subjects (Supplementary Figure S4). The samples were concentrated using Vivaspin 500 and were dried by evaporation using a SpeedVac centrifuge (Scan Speed Maxi Vac, Labogene, Denmark). The samples were then dissolved in a lysis buffer containing 8 M urea and 0.1 M Tris-HCl (pH 8.5) and quantified using the Bradford kit (Pierce, Rockford, IL, USA) with BSA as the standard, according to the manufacturer’s instructions. The samples were reduced by incubation with 5 mM Tris (2-carboxyethyl) phosphine (Pierce, Rockford, IL, USA) at 37 °C for 30 min with shaking and alkylated with 15 mM iodoacetamide (Sigma-Aldrich, St, Louis, MO, USA) at 25 °C for 60 min with shaking. The proteins in the samples were digested to peptides by mass spectrometry grade Trypsin Gold (Promega, Madison, WI, USA) and incubated at 37 °C for 16 h. Following digestion, the peptides from the pooled sample were fractionated by the OFF-gel fractionator (Agilent Technologies) based on isoelectric points of the peptides in the 12-well setup according to the manufacturer’s instructions. Individual samples did not undergo the OFF-gel fractionation.

### 4.3. HPLC-Triple 5600 Mass Spectrometry

The peptides were analyzed using the Nano-LC system Ekspert nLC415 (Eksigent Technologies, Dublin, CA, USA) equipped with AB SCIEX 5600 triple TOF mass spectrometer (AB SCIEX; Concord, Canada). The injection volume was 2 µL and the peptides were trapped on a Nano-LC trap column (0.5 mm × 350 µm; 3 µm; Eksigent Technologies) for 7 min through the mobile phase A, which consisted of 0.1% FA in 100% HPLC water. The separation of peptides was then conducted using an analytical column (150 mm × 75 µm; 3 µm; Eksigent Technologies), which was linked with a nanospray tip (PicoTip Emitter Silica Tip by New Objective, Woburn, MA, USA). The flow rate was set at 300 nL/min for 120 min with mobile phase B, which consisted of 0.1% FA in ACN and set with a linear gradient: 5%–40% for 105 min, 40%–90% for 0.5 min, 90% for 6 min, 90%–5% for 0.5 min, and 5% for 8 min. β galactose (50 mol) was used for autocalibration after every three injections. The Q-TOF conditions used were as follows: ion source gas and curtain gas were set to 12 and 25, respectively, ion spray voltage floating was set to 2200 V, and interface heater temperature was set to 150 °C.

### 4.4. Data-Dependent Analysis and SWATH Analysis

Using the information-dependent acquisition (IDA) mode, proteins in the samples were identified. Search parameters were as follows: TOF/MS survey was performed in the mass range of 250–2000 m/z. The top 10 parent ions with charges ranging from +2 to +5 and intensity greater than 100 cps were selected. The mass range of the product ions was scanned at 100 m/z up to 2000 m/z. The DIA mode was applied to the AB SCIEX 5600 triple TOF mass spectrometer for SWATH analysis. The TOF mass scan was performed from 250 m/z to 2000 m/z, and the product scan was performed from 250 m/z to 2000 m/z. The isolation width was 20 Da (1 Da is the window overlapping) and 53 windows were generated. Supplementary Table S3 provides information for all the peptides used for protein identification.

### 4.5. Database Exploration and Statistical Analysis

LC-MS/MS raw data were processed using the ProteinPilot v5.0 search engine (AB SCIEX) for the identification of peptides and proteins (Release April 2016). The parameters were set as follows: instrument, TripleTOF 5600; species, homo sapiens; Cys alkylation, iodoacetamide; digestion, trypsin allowing for 2 missed cleavages; false discovery rate (FDR) lower than 1%. We selected eight candidate peptides per protein with an FDR threshold lower than 1% and 99% peptide confidence, except for shared peptides and modified peptides. To conduct relative quantitative analysis, MarkerView was used. After the relative protein or peptide intensity of individual samples was extracted from Peakview, the intensity was normalized by the total sum area using MarkerView software. Normalized data were then used for principal component analysis (PCA) and *t*-tests to identify differential expression of proteins between ischemic stroke patients and healthy subjects. Among the identified proteins, differentially expressed proteins with statistical significance between ischemic stroke and healthy subjects were analyzed. The heat map, which is freely available at the web server (http://www.heatmapper.ca), showed the expression of differentially expressed proteins between individual samples. GeneGo Metacore (Version 6.29; GeneGo, MI, USA) was used for pathway analysis. GeneGo Metacore was used for assessing human protein–protein interaction as well as the construction of pathway maps, networks, and selection of major pathways related to the disease. The selected 13 candidate protein–protein interactions were analyzed using STRING. The classifier model was constructed in SPSS version 18 (SPSS Inc., Chicago, IL, USA).

### 4.6. Multiple Reaction Monitoring Using Triple Quadrupole Mass Spectrometry

Scheduled MRM analysis was carried out on a triple quadrupole linear ion trap mass spectrometer (AB SCIEX 5500 QTRAP) connected with an electrospray ionization (ESI) source. To select MRM ion pairs, we used the Skyline software, which assisted in the selection of appropriate parameters, including the MRM precursor, fragment ion, declustering potential (DP), and collision energy (CE). The precursor ion charge of the selected 13 peptides was set at +2 while the fragment ion charge was selected as 2 to +3. One precursor ion and three fragment ions per peptide were picked to select MRM Q1/Q3 ion pairs for the 13 peptides. We selected the MRM ion pair with the best linearity (r^2^ > 0.987) in the DP and CE of the same condition (Supplementary Table S4). Dot plots and OC curves were used to confirm the expression patterns and discriminatory power of the selected candidate proteins. A sample injection volume of 5 µL was used on an ACQUITY UPLC BEH C18 (2.1 × 150 mm, 1.7 µm; Waters) column connected to a Waters guard column (2.1 × 5 mm, 1.7 µm; waters). The analysis time was 30 min with 90% solvent A and 10% solvent B for 1 min, 85% solvent A and 15% solvent B for 19 min, 60% solvent A and 40% solvent B for 5 min, and 90% solvent A and 10% solvent B for 5 min at a flow rate of 0.25 mL/min. The mobile phase A and B were the same as mentioned in Section 4.6. MRM data results were processed by MultiQuant 2.0.2, supported by AB SCIEX.

## Figures and Tables

**Table 1:** The 13 candidate protein biomarkers related to risk factors and pathogenesis of ischemic stroke.

Accession No.	Protein Name	Risk Factors				Pathogenesis		
		Hypertension	Cardiovascular Disease	Diabetes	High Cholesterol	Immune Response	Oxidative Stress	Apoptosis
P00734	Prothrombin		√	√				
P00740	Coagulation factor IX		√	√				
P00747	Plasminogen							√
P02671	Fibrinogen alpha chain	√	√	√		√		√
P02751	Fibronectin		√	√		√		
P04004	Vitronectin		√	√		√		√
P04196	Histidine-rich glycoprotein		√			√		√
P07225	Vitamin K-dependent protein S				√	√		√
P07996	Thrombospondin-1	√	√	√	√	√	√	√
P09871	Complement C1s subcomponent					√		
P22352	Glutathione peroxidase 3	√	√	√			√	√

## Supplementary Materials

The following are available online at https://www.mdpi.com/2075-4418/10/5/340/s1, Figure S1: Unsupervised Principal Component Analysis (PCA) of the label free quantification proteomics data from healthy controls and stroke patients, Figure S2: Protein interaction network of candidate proteins, Figure S3: Overall workflow for stroke marker identification, Figure S4: MS spectra of 12 sample fraction that were used to bulid the SWATH library, Table S1: Total protein identification of healthy controls and stroke patients, Table S2: Demographic information of the stroke patients., Table S3: The information of peptides identified by SWATH-MS, Table S4: Candidate transition between healthy controls and stroke patients.
