# Supplementary material for: Proteomics-Based Identification of Diagnostic Biomarkers Related to Risk Factors and Pathogenesis of Ischemic Stroke
Source: Diagnostics (Basel). 2020 May 25;10(5):340. doi: 10.3390/diagnostics10050340 (PMC7278009; doi:10.3390/diagnostics10050340)
Supplement: Supplementary file 1 [file diagnostics-10-00340-s001.zip › Supplementary data/Fig. S1~4.docx]

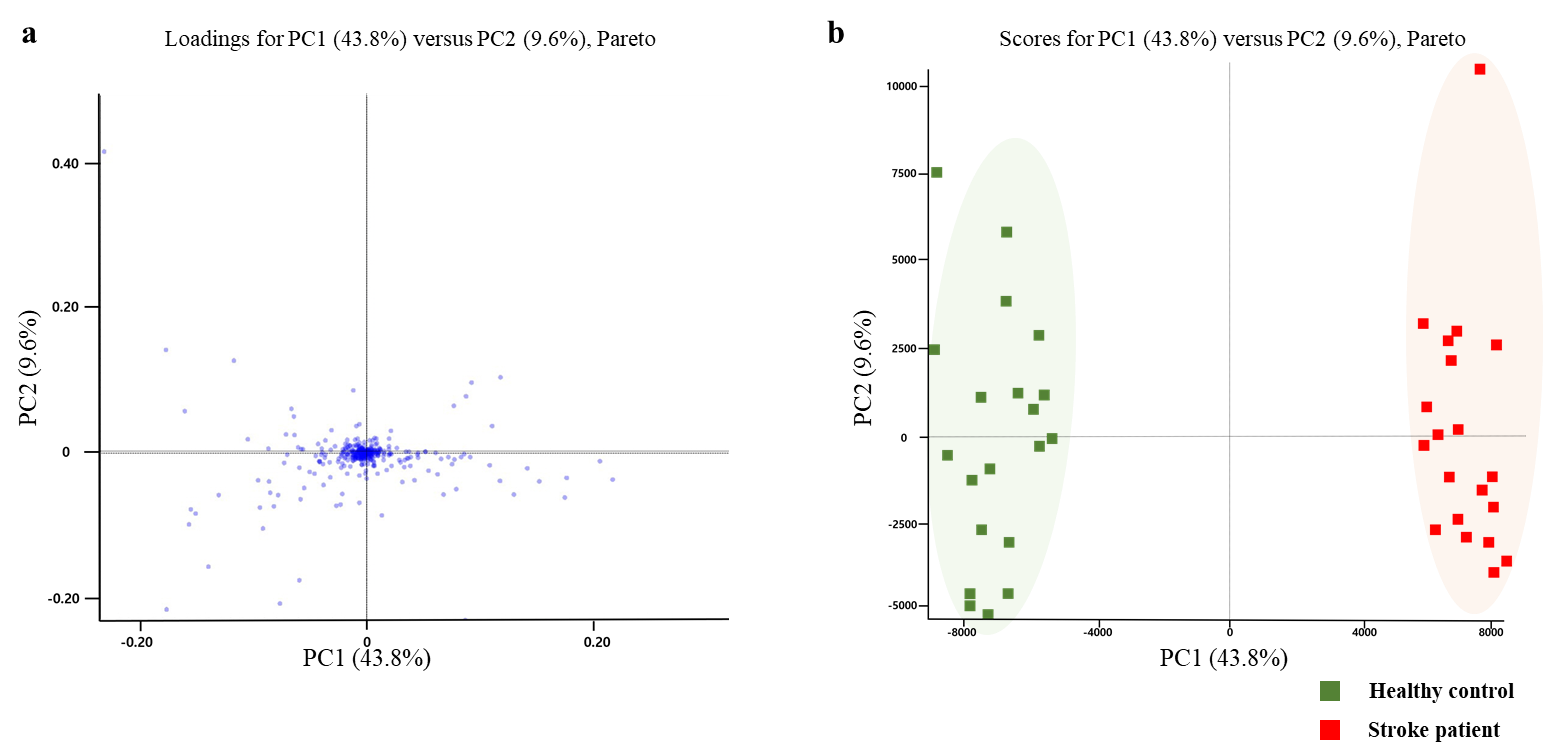


**Fig. S1.** Unsupervised Principal Component Analysis (PCA) of the label free quantification proteomics data from healthy controls and stroke patients, (A) The proteins contributed to distinguish healthy controls and stroke patients. (B) The classification of healthy controls and stroke patients was performed based on the score scatter plot.


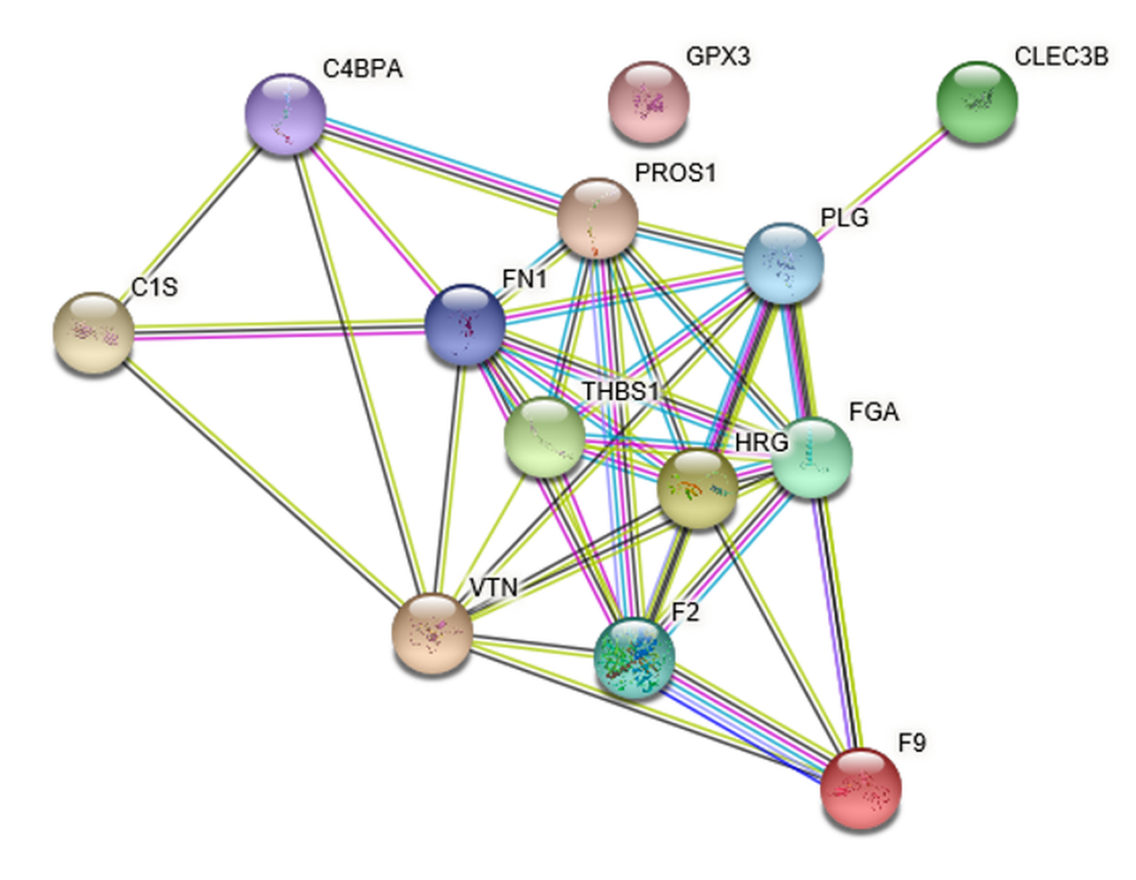


**Fig. S2.** Protein interaction network of candidate proteins, The lines that have different color are shown in various biological process. Most of proteins are linked with other proteins. F2, prothrombin; F9, coagulation factor IX; PLG, plasminogen; FGA, fibrinogen alpha chain; FN1, fibronectin; C4BP4, c4b-binding protein alpha chain; VTN, vitronectin; HRG, histidine-rich glycoprotein; CLEC3B, tetranectin; PROS1, vitamin K-dependent protein; THBS1, thrombospondin 1; C1s, complement C1s subcomponent; GPX3, glutathione peroxidase 3.

**
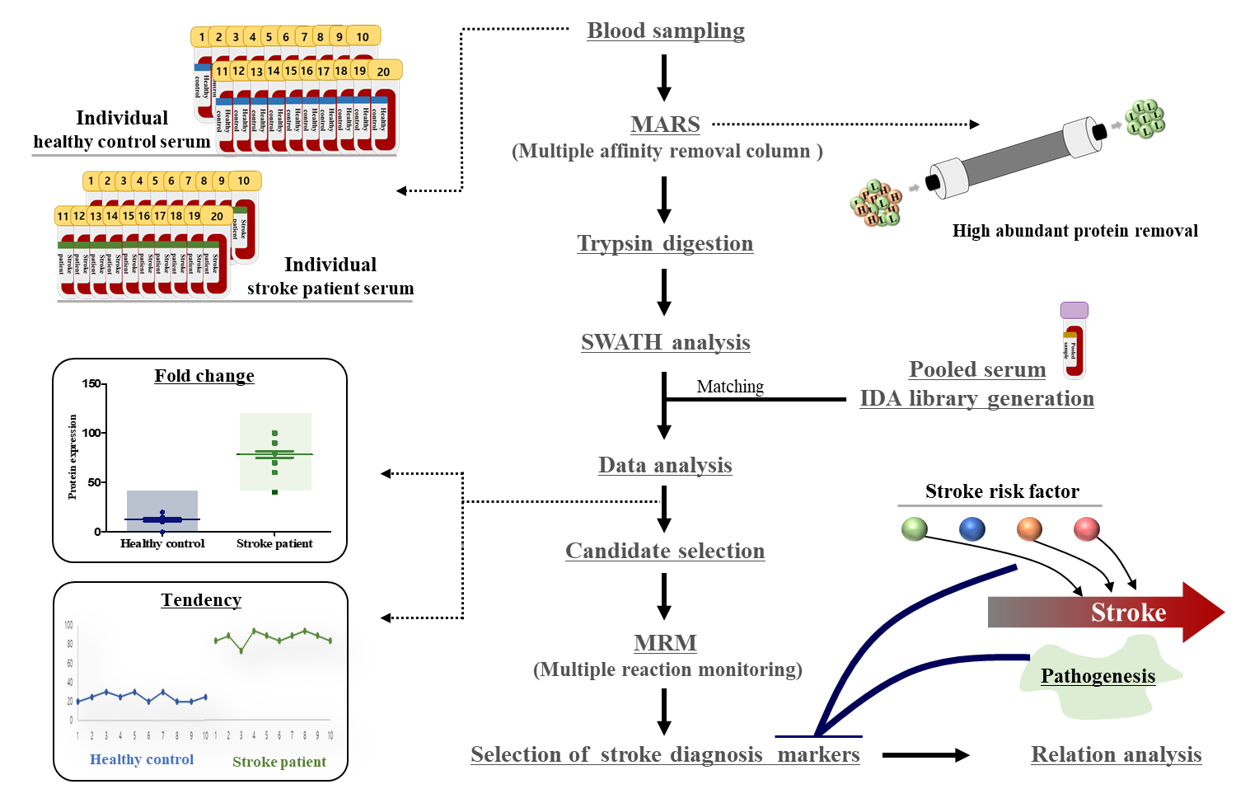
**

**Fig. S3.** Overall workflow for stroke marker identification, Blood samples were collected from each healthy control and stroke patients. Multiple affinity removal system (MARS) liquid chromatography (LC) column was used to deplete high abundant proteins in all samples. The SWATH-analysis, that is independent analysis, was performed using 20 healthy control and 20 stroke patient samples for quantification analysis. The candidate proteins were selected from SWATH data and verified using MRM. Characteristic of selected stroke diagnosis candidate markers were associated with stroke risk factors and pathogenesis.


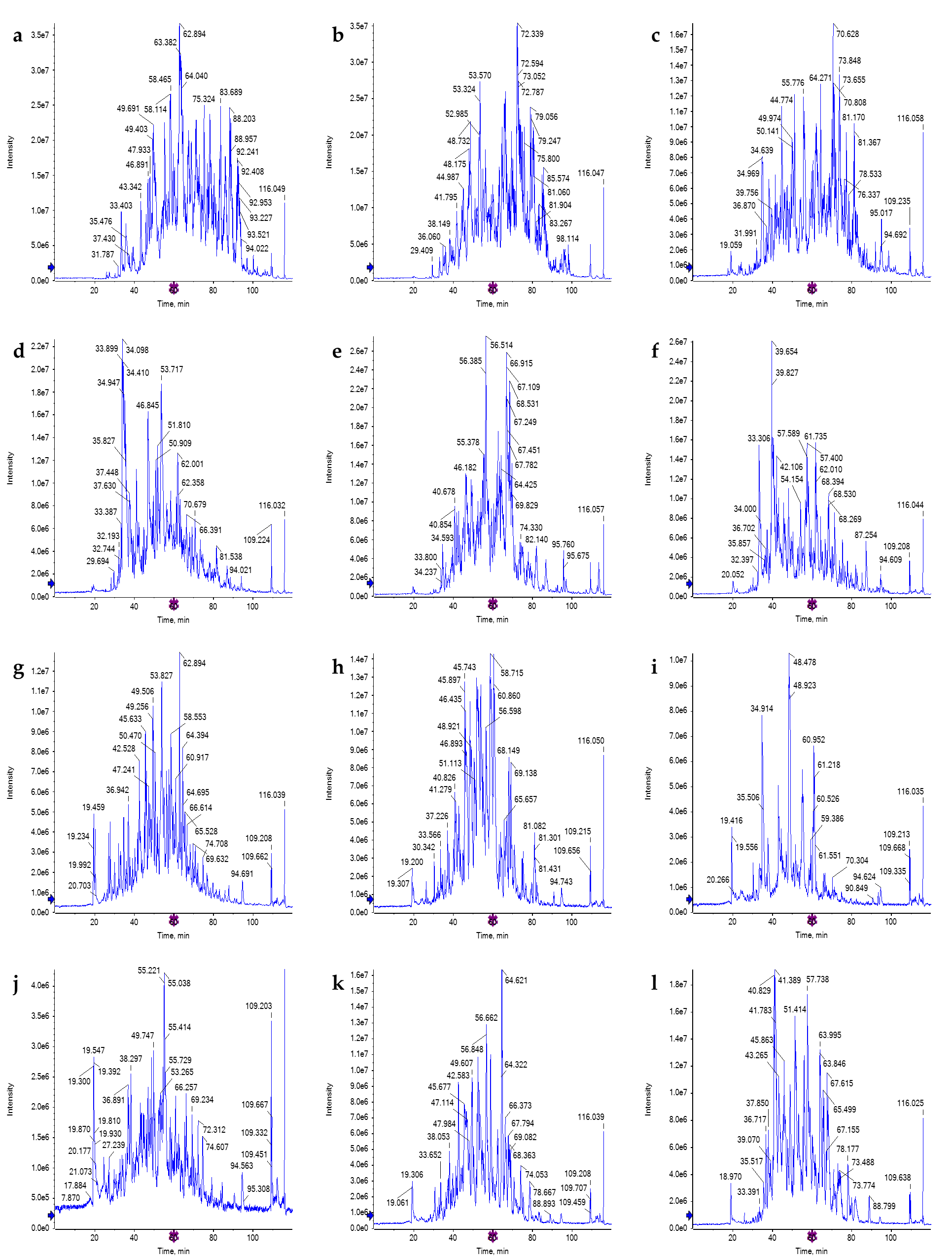
**Fig. S4.** MS spectra of 12 sample fraction(a~l) that were used to bulid the SWATH library.
